# Cardiac adverse events of PD-1 and PD-L1 inhibitors in cancer protocol for a systematic review and network meta-analysis: A protocol for systematic review

**DOI:** 10.1097/MD.0000000000018701

**Published:** 2020-01-31

**Authors:** Deting Han, Jianyong Dong, Honglin Li, Tao Ma, Wenjun Yu, Lucheng Song

**Affiliations:** a Shandong University of Traditional Chinese Medicine; b Gao Xin Branch of Jinan Stomatological Hospital; c The First Hospital Affiliated with Shandong First Medical University (Shandong Provincial Qianfoshan Hospital), Jinan, China.

**Keywords:** cancer, cardiac adverse events, network meta-analysis, PD-1 and PD-L1 inhibitors, protocol, systematic review

## Abstract

**Introduction:**

Programmed cell death 1 (PD-1) and programmed cell death ligand 1 (PD-L1) inhibitors have been increasingly used in the treatment of cancer. Immunosuppressive therapy can control the cancer well and is suitable for the moderate to severe diseases. However, according to clinical observation, immune-related cardiac adverse events against PD-1or/and PD-L1 are inevitable, but generally reversible. Understanding the cardiac adverse events of PD-1 or/and PD-L1 inhibitors is crucial to improve the anti-cancer efficacy and ensure the life safety of patients. The variability of cardiac adverse events between different immunosuppressants and different cancers is not clear.

**Methods and analysis:**

This protocol established in this study has been reported following the Preferred Reporting Items for Systematic Review and Meta-Analysis Protocols. We will search the following electronic bibliographic databases: PubMed, Cochrane Library, EMBASE databases and ClinicalTrials.gov from their inception to December 2019. We will use a combination of Medical Subject Heading, and free-text terms with various synonyms to search based on the Eligibility criteria. We will include RCTs on PD-1 or/and PD-L1 inhibitors therapy to analyze. In addition, our study will include some clinical trials. All relevant RCTs will be included, such as early phase I/II, phase III experimental trials, prospective and retrospective observational studies. According to the inclusion and exclusion criteria outlined above, the full texts of each eligible study will be retrieved for further identification by one reviewer. Two authors will screen the titles and abstracts of all records retrieved in above electronic databases independently to find potentially eligible reviews. Data will be extracted by 2 reviewers independently using a pre-designed data extraction form. The other reviewer will validate data. I-square (I^2^) test, substantial heterogeneity, sensitivity analysis and publication bias assessment will be performed accordingly. For our network meta-analysis, we will use Stata 15.0 and WinBUGS 1.4.3.

**Ethics and dissemination:**

Ethics approval and patient consent would be not required because the data of this network meta-analysis mainly are obtained from existing resources. This network meta-analysis will be published in a peer-reviewed journal.

**PROSPERO number:**

CRD42019142865

## 1 Introduction

Programmed cell death-1 (PD-1) and cytotoxic T-lymphocyte antigen-4 (CTLA-4) are included immune checkpoint inhibitors (ICIs),^[1]^ and programmed cell death-1 (PD-1) is the most effective immune checkpoint blocker developed in recent years.^[2]^ Over the past 10 years, ICI has been recognized as one of the most important breakthroughs in cancer therapy.^[3]^ There are Standards guidelines for the clinical trails of adverse events treated with PD-1 and PD-L1 inhibitors. But according to the report, there is a large amount of variability in drug and dose schedule, and adverse event reporting in cancer in publications.^[4]^ Based on pooled data from pivotal trials as reported by the European Medicines Agency, the present paper reviews incidences and kinetics of onset and resolution of immune-mediated “adverse events of specific interest” (AEOSI) of both approved PD-1 inhibitors nivolumab and pembrolizumab. In general, the severity of AEOSI is mild to moderate (grade 1–2); the frequency of immune-mediated but also idiopathic grade 3–4 adverse drug reactions is 62% for any event term.^[5]^ Immune-checkpoint inhibition has revolutionized the treatment of various malignancies including melanoma, non-small-cell lung cancer, renal cell carcinoma, Hodgkin lymphoma, bladder cancer, head and neck cancer, gastric cancer, liver cancer.^[6]^ In patients with that cancers, anti-PD-1 or anti–PD-L1 therapy unleashes the immune system, and generates powerful antitumor T cell responses.^[7]^ Anti-PD-1 or anti–PD-L1 therapy drugs include monoclonal antibodies directed at both PD-1 (nivolumab, cemiplimab, and pembrolizumab) and the PD-L1 (avelumab, atezolizumab, and durvalumab).^[8]^ A prior clinical study showed that ipilimumab and nivolumab dramatically improved response rates in patients with advanced melanoma from 19% with ipilimumab alone to 58% with the combination.^[9]^ Nivolumab also increased the survival of patients with previously untreated melanoma, and was therefore approved in December 2014 for the treatment of unresectable or metastatic melanoma by FDA.^[10]^ These drugs all work by blocking the PD-1 or PD-L1 immune checkpoint pathway to reactivate T cell-mediated antitumor immunity.^[11]^

However, immunotherapy is associated with several immune-related adverse events,^[12]^ especially with combination immunotherapies, have led to hesitation among cancer immunotherapy patients.^[13]^ Their side effects are equally fascinating as immune-related adverse events (irAE) have been described in almost all organs,^[14]^ including the cardiovascular system. These adverse events would manifest either slowly over time, leading to significantly increased long-term cardiovascular morbidity and mortality, or acutely in the days to weeks after treatment.^[15]^ The cardiac adverse events is particularly undefined for novel therapies,^[16]^ and their application is associated with a spectrum of immune-related adverse events.^[17]^

ICI associated cardiac adverse events resulted from misdirected stimulation of the immune system^[18]^ can manifest in a variety of ways, including fulminant lymphocytic myocarditis, supraventricular and ventricular arrhythmias, pericardial disease, and even Takotsubo-like cardiomyopathy.^[19]^ Any of that was assumed to be included in the monitoring cost for the progressed disease state.^[20]^

The events incidences have been reported in patients treated with ipilimumab and combination of ipilimumab and nivolumab, myocardial fibrosis, left ventricular dysfunction, Takotsubo cardiomyopathy and late-onset pericarditis.^[3,21]^

The use of checkpoint inhibitors may be associated with the development of cardiac disease.^[22]^ Immune-mediated myocarditis is characterized by fulminant progression,^[23]^ and a global cardio-immuno-oncologic assessment seems important to detect potential toxicities of newer immunotherapies, also acknowledging that these drugs are often combined with cardiotoxic kinase inhibitors.^[24]^

Cardiac adverse events have been widely reported. However, substantial variations exist in cancer type, drug and dosing schedule, and AEs reporting criteria in these studies. In addition, meta-analysis of cardiac adverse events by anti-PD-1 or anti–PD-L1 drugs has not been reported comprehensively. Therefore, it is of great significance to clarify the cardiac adverse events caused by different drugs to guide clinical treatment, and to improved survival of patients.

## 2 Objective

This network meta-analysis (NMAs) will explore the cardiac adverse events of PD-1 and PD-L1 inhibitors in cancer patients.

## 3 Methods

The protocol follows the Preferred Reporting Items for Systematic Review and Meta-analysis Protocols.^[25]^ This review has been registered on the PROSPERO, where dates, changes, and rationales are tracked if protocol revisions occur.

### 3.1 Eligibility criteria

#### 3.1.1 Types of studies

We will include RCTs on PD-1 or/and PD-L1 inhibitors therapy to analyze. In addition, our study will include some clinical trials. All relevant RCTs will be included, such as early phase I/II, phase III experimental trials, prospective and retrospective observational studies. However, it will be necessary to exclude the reviews, meta-analysis, case reports, and studies with insufficient data. Only studies in English will be considered.

#### 3.1.2 Types of participants

Patients enrolled in randomized controlled trials testing anti-PD1/PD-L1 targeted therapies. Ignoring the type of cancer and previous treatment therapy, such as surgery, radiation, and chemotherapy etc.

#### 3.1.3 Types of interventions

At least 1 of the study arms consisting of nivolumab or pembrolizumab or avelumab or atezolizumab or cemiplimab or camrelizumab or durvalumab. Studies of ICI combining with radiotherapy, cell vaccines, small molecule inhibitors, and immunotherapy with IL-2 or interferon will be excluded.

#### 3.1.4 Types of outcome

The primary outcome will be relative cardiac adverse events treated with PD-1or/and PD-L1 inhibitors, such as reported tabulated data on Heart failure, myocardial infarction, arrhythmia and so on in overall population. Moreover, the types, numbers and time of cardiac adverse events will be regarded as indispensable data to analyze.

### 3.2 Information sources

The search will be conducted in the following electronic bibliographic databases: PubMed, Cochrane Library, EMBASE databases and ClinicalTrials.gov from their inception to December 2019. Table 1 shows the details of search strategy for PubMed.

**Table 1:**
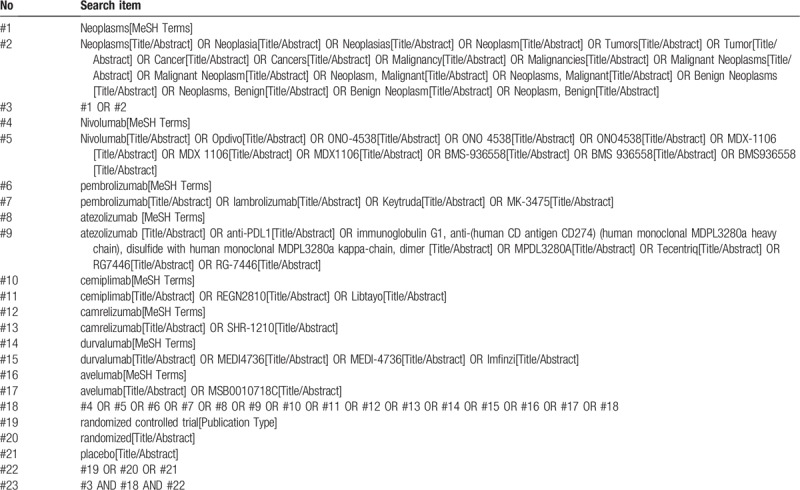
Details of the search strategy for PubMed.

### 3.3 Search strategy

We will use a combination of Medical Subject Heading, and free-text terms with various synonyms to search based on the Eligibility criteria. There will be no restrictions on author, date limit, country, publication status, or year of publication.

### 3.4 Study records

#### 3.4.1 Study selection

Literature search records from electronic databases will be imported into the EndNote X9 literature management software. Two authors will screen the titles and abstracts of all records retrieved in above electronic databases independently to find potentially eligible reviews. According to the inclusion and exclusion criteria outlined above, the full texts of them will be retrieved for further identification. Any disagreement will be resolved by discussion or by consultation with a third author.

#### 3.4.2 Data extraction

The following data will be extracted by 2 reviewers: First author, Participants, Country, Publication time, Study design, Trial name, Trial phase, Cancer type, PD-1and PD-L1 inhibitors used, Doses schedules, Types of adverse event, All the results etc. Two reviewers will make a data extraction form according to the above items and will extract the required data from each full text for further identification. The other reviewer will validate data.

### 3.5 Risk of bias assessment

We will use the Cochrane risk bias tool, which consists of the random sequence generation, allocation concealment, blinding of investigators and subjects, blinding of study outcome evaluation, outcome data integrity, selective reporting of study results and other sources of bias.^[26]^ While each entry is based on the risk of bias assessment criteria will be made 3 levels: “low risk of bias,” “unclear risk of bias,” “high risk of bias.”

### 3.6 Data synthesis

First, we will use a random-effects meta-analysis, with standardized mean differences for continuous outcomes, and use odds ratio (OR) or risk ratios (RR) to compare dichotomous variables. All the results will be reported with 95% confidence intervals (CI). RR or OR, 95% confidence intervals and two-sided *P* values will be pooled for dichotomous data to estimate using the Mantel–Haenszel method for each outcome.

Second, I-square (*I*^2^) test will be used to assess the impact of Heterogeneity between the studies using the statistic. We will consider an *I*^2^ value greater than 50% as being indicative of substantial heterogeneity.

Third, we will use stratified meta-analyses to explore heterogeneity in effect estimates according to study quality; study populations; the logistics of intervention provision; and intervention content. We will also assess evidence of publication bias.

Forth, we will conduct sensitivity analyses based on study quality.

Fifth, we will perform a Bayesian network meta-analysis model for each outcome to estimate the overall treatment effects.

Sixth, in our network meta-analysis, we will use Stata 15.0 and WinBUGS 1.4.3.

#### 3.6.1 Subgroup analysis

If possible, subgroup analyses will be performed according to the intervention of study design: anti-PD-1 or/and anti-PD-L1 vs ipilimumab, anti-PD-1 or/and anti-PD-L1 vs chemotherapy, anti-PD-1 or/and anti-PD-L1 vs radiation therapy and nivolumab plus ipilimumab vs ipilimumab. In addition, we will try to use age, gender, race, countries and regions to explain the sensitivity and heterogeneity.

#### 3.6.2 Publication bias

A comparison-adjusted funnel plots graph will be drawn by the Stata 15.0 software to assess the potential publication bias. In the funnel plot, the magnitude of the action is the horizontal axis, and the measurement of its precision, such as the sample size, is the vertical axis. With the increase of sample size, the random change of action decreases. Thus, if there is no publication bias, the data obtained from each study will be distributed symmetrically on the graph in an inverted funnel. On the contrary, the asymmetric inverted funnel pattern indicates the existence of research sample bias.

### 3.7 Quality assessment of evidence

Two evaluators to assess quality independently. Any discrepancy would be discussed, or the third evaluator shall make a decision. The Grading of Recommendations Assessment Development and Evaluation (GRADE) system classifies the quality of evidence into 4 levels: high, medium, low, and very low.^[27]^ Otherwise, guideline development tool (GDT) will be used to conduct this process.

## 4 Discussion

This network meta-analysis will provide a detailed summary and analysis of cardiac adverse events associated with anti-PD-1and PD-L1 drugs. Anti-PD-1 and anti-PD-L1 drugs as an Immunotherapy, are overall less toxic than standard chemotherapy on treatment of previously untreatable malignancies. However, many other immune-related cardiac adverse events have been found in the clinical, such as myocarditis, heart failure, arrhythmia, myocardial hypertrophy, etc. Reporting cardiac adverse events during the treatment of tumor by anti-PD-1and PD-L1 drugs, providing a complete event profile and event spectrum, and to explore the safety of anti-PD-1and PD-L1 drugs will be considered essential. Therefore, a high suspicion of cardiac adverse events is warranted as timely diagnosed and treated, and can avoid life threatening complications.^[14]^

However, there are still limitations in this network meta-analysis. Retrospective studies are not only inherently low quality, but also increase potential publication bias. In addition, some studies may be of poor quality for decreasing the significance of the results in network meta-analysis. Moreover, heterogeneity among different studies may affect the final results of this study.

Evaluation of safety and efficacy will help us to choose better treatment. It is necessary to conduct larger scale registries and longitudinal studies of patients receiving anti-PD-1and PD-L1 drugs and prospective. According to that, the best immunosuppression regimen for different types of cardiac adverse events will be determined.

Recommended risk minimization procedures include a patient alert card and a physician education summary in order to keep rates of occurrence and worsening as low as possible.^[5]^ Anti-PD-1 therapy should be maintained in case of grade 1 toxicities; a therapy restart after recovery from grade 2 events or higher grade cardiac adverse events is highly recommended to allow therapy continuation. The early detection of AEOSI and close clinical monitoring are essential for successful management. Therefore, it is imperative to conduct further study on immune-related cardiac adverse events. Nonetheless, we hope that our findings will assist patients, clinicians and healthcare policymakers to make a better choice of treatments in cancer, and encourage further research to enhance clinical decision-making.

## Author contributions

Deting Han and Jianyong Dong are joint first authors.

Deting Han, Jianyong dong and Lucheng Song designed the study.

Honglin Li, Tao Ma and Wenjun Yu managed the data. Deting Han drafted the manuscript.

Lucheng Song is the guarantor of the review, who is contributed to the interpretation of the results and critical revision of the manuscript for important intellectual content and approved the final version of the manuscript.
